# Acute shortening of upper extremity in orthopaedic patients: a scoping review

**DOI:** 10.1007/s00068-025-02904-9

**Published:** 2025-06-11

**Authors:** Zhijian Sun, Gang Liu, Nikolaos K. Kanakaris, Ting Li, Xinbao Wu, Peter V. Giannoudis

**Affiliations:** 1 Department of Orthopaedics Trauma, Beijing Jishuitan Hospital, Capital Medical University, Beijing, 10035 China; 2 Academic Department of Trauma and Orthopaedic Surgery, School of Medicine, University of Leeds, Clarendon Wing, Floor D, Great George Street, Leeds General Infirmary, Leeds, LS1 3EX UK

**Keywords:** Acute shorting, Upper extremity, Amputation, Fracture non-union

## Abstract

**Purpose:**

Acute shortening of the upper extremity could be used in patients with segmental bone loss or large soft tissue defects. The study aimed to review available evidence about acute shortening procedures of the upper extremities to evaluate the common indications and tolerable shortening length.

**Methods:**

All clinical studies involving acute shortening procedures of the upper extremity were considered eligible for inclusion. PubMed, Embase, Web of Science and Cochrane Library for English-language articles from inception to December 2024 were searched. Two reviewers independently charted data from each eligible article.

**Results:**

Out of 730 studies screened, 35 articles met the inclusion criteria. There were 24 case series and 11 case reports containing 355 acute shortening procedures. Acute shortening was reported in 12 articles for amputation, 15 for fracture nonunion, 2 for fresh or delayed fracture, 2 for tumor, 2 for brachial plexus injury and 2 for forearm deformity. The maximum shortening for replantation was 10 cm in the forearm and 12 cm in the humerus. Shortening up to 2.9 cm and 8 cm for fracture nonunion were reported for the forearm and humerus, respectively.

**Conclusions:**

The main indications for acute shortening of the upper limb were amputation for replantation and fracture nonunion. Aggressive bony shortening was recommended in the setting of amputations. Controversies existed about the tolerated shortening length for both the forearm and humerus.

**Supplementary Information:**

The online version contains supplementary material available at 10.1007/s00068-025-02904-9.

## Introduction

Acute shortening of extremities during surgery is useful in clinical situations such as amputation, deformity correction, and bone defects caused acutely or secondarily following fracture nonunion or tumor resection [1–5]. It also remains an important principle in replantation surgeries [6, 7]. Bone shortening of upper limbs enables aggressive debridement and tensionless primary neurovascular repair whilst facilitating appropriate wound closure [2, 8]. Moreover, it simplified bone stabilisation, which reduced the ischemic time in replantation procedures [9]. In large bone defects caused by infection, nonunion, tumor resection or deformity osteotomy, acute shortening allows bony apposition, facilitating bone union and reducing the need for bone grafting [1, 3–5]. 

Length discrepancy following bone shortening of the upper extremity compared with the lower extremity is considered more tolerable without significant function compromise and cosmetic concerns [10]. Thus, lengthening seldomly is performed at the second stage of nonunion revision surgery [11–13]. 

Nevertheless, acute shortening would probably influence strength and range of motion since the optimum length of muscles and the biomechanic working length of the anatomic levers are altered [14–17]. In a computational model study, triceps muscle force reduction after shortening of the distal humerus was observed in a range between 11 and 63% depending on the length of shortening and position of the elbow [17]. In cadaveric studies, forearm shortening was found to be related to a reduction of the pronation and supination movements [14, 15]. In the clinical setting, the functional outcomes were affected by such parameters as neurovascular injuries in amputation, tortuous blood vessels after acute shortening (leading to distal ischemia in non-anastomosis patients) and soft-tissue scarring [9, 18–21] To date, the indications and the length of the accepted shorting of the upper extremity have not been well established.

Thus, the present scoping review was conducted to systematically map the quantity, design and characteristics of available literature on acute shortening procedures of the upper extremities. The following research questions were formulated: What are the common indications for acute shortening of the upper extremity? And what was the maximum tolerable shortening length without significantly compromising limb circulation, function and cosmetic appearance?

## Methods

### Protocol and registration

A review protocol was drafted according to the PRISMA guidelines extension for Scoping Reviews (PRISMA-ScR) and was reviewed by all the authors [22, 23]. The final protocol was registered prospectively with the Open Science Framework on January 7, 2025 (https://osf.io/f3re7).

### Eligibility criteria

For the review, papers required to include acute shortening procedures of the humerus or forearm. The following criteria needed to be fulfilled: (1) peer reviewed publications written in English, (2) involving patients who received acute shortening surgeries of forearm or humerus for any indication. Studies were excluded in cases of (1) gradual shortening of the upper extremity, (2) shortening of only one of the two bones of the forearm, or (3) wedge/doom osteotomies of either the humerus or the radius.

### Information sources and search strategy

The following bibliographic databases were searched from inception to December 2024 to identify potentially relevant papers: PubMed, Embase, Web of Science and Cochrane Library. The first author drafted the search strategies and further refined them through team discussion. The following keywords were used: acute shortening, upper extremity, forearm, humerus, acute bone loss, mangled extremity injury, amputation, infection debridement, fracture nonunion, tumor resection and deformity osteotomy. The final search strategy was listed in the supplemental materials. The final search results were exported to NoteExpress, and duplicate papers were removed.

### Selection of sources of evidence

**Fig. 1:**
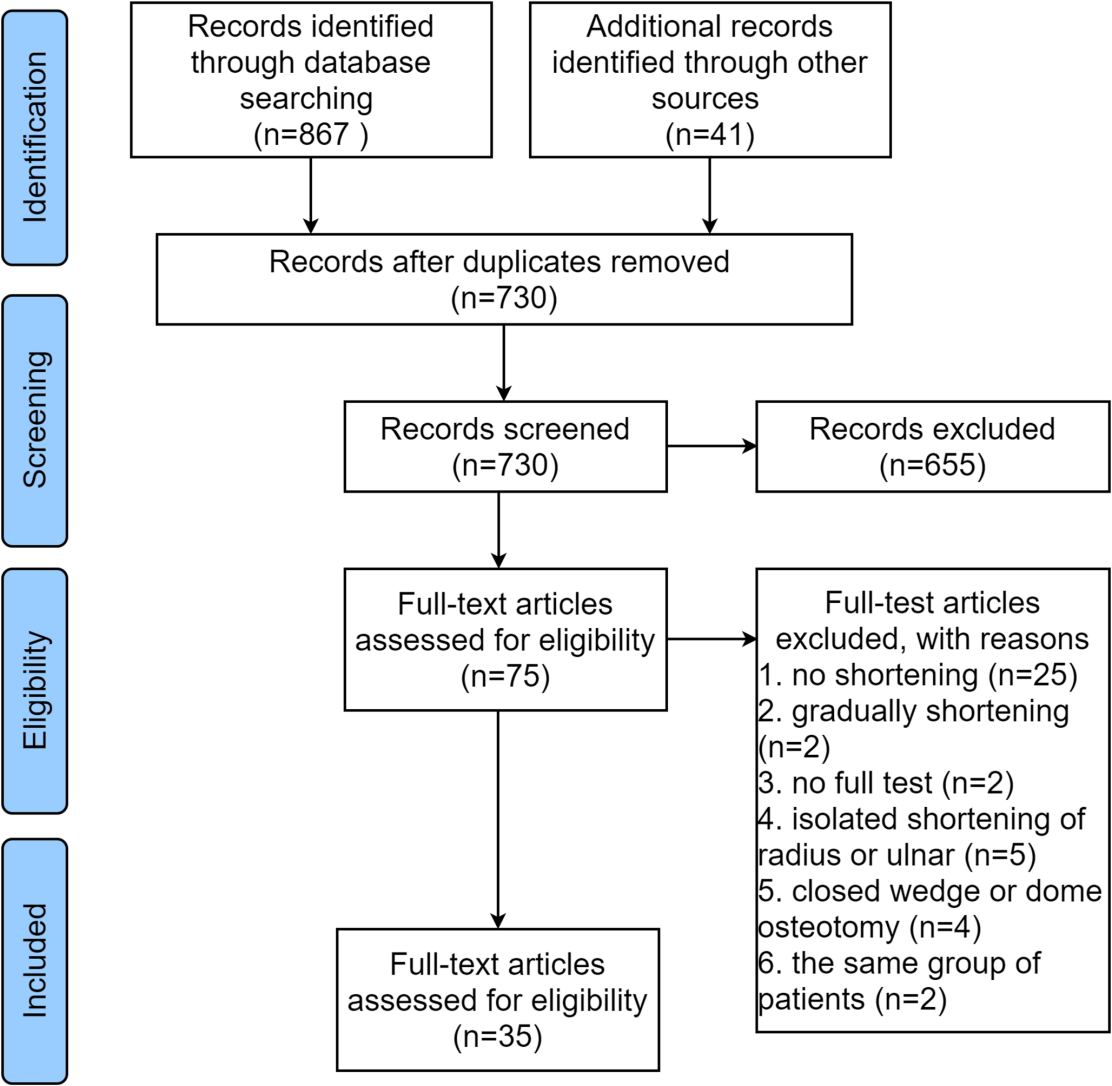
PRISMA flow diagram of the scoping review included and excluded

Two authors (Z.J. Sun and G. Liu) first worked in pairs to evaluate the titles and abstracts, and irrelevant articles were excluded. Then, the whole text was reviewed, and relevant articles were identified. Reference lists of the included articles or relevant reviews were further examined to identify additional relevant material. Disagreements were resolved by team discussion. The process of selection through stepwise exclusion is illustrated in Fig. 1.

### Data charting process

The first author developed a data charting form, which was revised and approved by the review team to determine which variables to extract. Two reviewers (Z.J. Sun and G. Liu) independently charted data from each eligible article. Any disagreements were resolved through discussion between the two reviewers while the senior author (P.V. Giannoudis) approved the final decision. Data charting was implemented using Microsoft Excel. Since this study was a scoping review and was conducted to provide an overview of the existing evidence regardless of methodological quality, risk of bias assessment was not carried out.

### Data items

Studies were firstly divided into those referring to forearm or humerus shortening. Data items include author, year of publication, study location, study type, diagnosis, number of patients enrolled, patients’ age, length of shortening, fixation instruments, type of bone graft, union rate, complication rate, reoperation rate and clinical outcomes.

### Synthesis of results

The reported diagnosis in all included cases was used to identify the main indications of the acute shortening surgery. The mean and range of shortening lengths were calculated as recorded. Main outcomes, such as union rate, complications, clinical outcomes and need for further surgery, were also retrieved.

## Results

### Selection of sources of evidence

A total of 730 articles were identified after duplicates were removed, as evident in the PRISMA flowchart (Fig. 1). In total, 35 articles met the inclusion criteria and were eligible for final analysis [6, 7, 9, 12, 18–21, 24–51]. There were 14 articles reporting forearm/elbow shortening [6, 9, 18–21, 25, 28, 30, 34, 38, 49–51]., and 18 reporting on humerus shortening [12, 24, 26, 27, 29, 31–33, 35–37, 39–41, 43, 44, 47, 48]. Three studies reported on both forearm and humerus shortening [7, 42, 46]. 

### Characteristics of the source of evidence

The 35 included articles were conducted across 13 countries, which were China (*n* = 6) [32, 33, 35, 44, 46, 49], UAS (*n* = 5) [25, 29, 31, 36, 40], India (*n* = 5) [19, 28, 39, 41, 42], Poland (*n* = 3) [26, 50, 51], UK (*n* = 3) [12, 20, 21], Turkey (*n* = 3) [7, 24, 27], Switzerland (*n* = 2) [6, 18], Austria (*n* = 1) [48], Brazil (*n* = 1) [47], Egypt (*n* = 1) [37], Korea (*n* = 1) [9], Pakistan (*n* = 1) [43], Singapore (*n* = 1) [30], South Africa (*n* = 1) [34], and Vietnam (*n* = 1) [38]. There were 24 retrospective case series [6, 7, 9, 12, 18, 24, 25, 27, 29, 31–33, 37–44, 46, 48, 50, 51]., and 11 case reports [19–21, 26, 28, 30, 34–36, 47, 49]. and no comparative studies.

Acute shortening surgeries were performed in both children [28, 42, 48, 51] and adult patients [7, 9, 12, 18–21, 24, 26, 27, 29–31, 33–44, 47–51] with an age range of 3 to 81 years. A total of 354 patients and 355 acute shortening procedures were altogether included in these 35 studies.

### Results of individual sources of evidence

Overviews and the main results of the included studies are illustrated in Tables 1 and 2.

**Table 1:** Extracted data displaying the study design and characteristics of acute shortening procedures of the forearm and elbow

Reference, first author, date	Country	Article type	Diagnosis	No. of enrolled patients	Age (yr, range)	Length of shortening (cm, range)	Fixation instruments	Type of bone graft	Union rate	Complication rate	Reoperation rate	Clinical function
Srikanth et al., 2024	India	Case report	Nonunion	1	43	2	Plates	Local autograft	100%	0	0	DASH: 5.8
Kim et al., 2023	Korea	Case series	Amputation	20	46 (25–68)	2.97 (0–7)	Plates/external fixator/rush pin	None	100%	NR^*^	NR	Chen’s classification I: 3, II: 4, III: 3, IV: 4
Young et al., 2018	South Africa	Case report	Amputation	1	22	2	Plate	None	100%	0	0	Chen’s classification: II
Ng et al., 2016	Singapore	Case report	Basal cell carcinoma of elbow	1	68	9	Plate	Local autograft	100%	0	200%	No functional score reported
Maheshwar et al., 2015	India	Case report	Nonunion of ulna	1	7	2.5	Plate/rush nail	Radial tubular graft	100%	0	0	No functional score reported
Burgess et al., 2012	USA	Case series	Wrist flexion deformity	6	NR	(1.5–2.5)	Steinman pin	None	NR	83.3% (5/6)	83.3% (5/6)	No functional score reported
Leclère et al., 2012	Switzerland	Case series	Amputation	11	43.4 (19–76)	4.3 (0–7)	Plate	None	100%	45.5% (5/11)	UC^**†**^	Chen’s classification I: 4, II: 3, III: 2, IV: 1
Sugun et al., 2009	Turkey	Case series	Amputation	13	29.6	3.8	Plate/K-wire/external fixator	None	NR	UC	UC	Chen’s classification I: 4, II: 5, IV: 4
Hoang et al., 2009	Vietnam	Case series	Amputation	10	21.2 (14–42)	3.2 (2.5–4)	K-wire/cerclage/plate/external fixator	None	100%	NR	80% (8/10)	Chen’s classification I: 1, II: 3, III: 3, IV: 3
Domanasiewicz et al., 2008	Poland	Case series	Volkmann contracture	15	17(3–54)	2.6(1.5–4)	Rush intramedullary nailing	Autograft	100%	26.7% (4/15)	13.3% (2/15)	Dexterity improvement of 10-grade scale: 7.13
Sabapathy et al., 2007	India	Case series	Amputation	17	28.7 (12–56)	7.5 (5–10); Proximal row carpectomy in 4 patients	K wire or plate	None	100%	23.5% (4/18)	88.2% (15/17)	Chen’s classification I: 3, II: 8, III: 4, IV: 2
Jabłecki et al., 2006	Poland	Case series	Amputation	48	34.2(16–64)	2.3(1.5–4.5) 4 patients without fractures	Intraosseous nails/wire loop	NR	NR	UC	UC	Chen’s classification I: 5, II:22, III: 14, IV: 7
Sharma et al., 2004	UK	Case report	Ulnar nonunion	1	19	2.9	Plate	None	100%	0	0	No functional score reported
Chuang et al., 2001	China	Case series	Amputation	12	NR	5 (4–6.5)	NR	NR	NR	UC	UC	No functional score reported
Chauhan et al., 1995	UK	Case report	Acute comminuted fracture	1	51	1.5	Plate	Iliac crest	100%	0	0	No functional score reported
Axelrod et al., 1991	Switzerland	Case series	Amputation of upper extremity	10	UC	3 (1–6)	Plate/external fixator/K-wire	None	UC	UC	UC	Chen’s classification: UC
Chen et al., 1991	China	Case report	Amputation	1 (bilateral)	17	5 (right)/6 (left)	Steinmann pins/retention wires	None	0	300% (3/1)	300% (3/1)	No functional score reported

*NR Not report; *† UC* Unable to calculate

**Table 2:** Extracted data displaying the study design and characteristics of acute shortening procedures of the humerus

Reference, first author, date	Country	Article type	Diagnosis	No. of enrolled patients	Age (yr, range)	Length of shortening (cm)	Fixation instruments	Type of bone graft	Union rate	Complication rate	Reoperation rate	Clinical function
Nieboer et al., 2024	USA	Case series	Supracondylar nonunion	28	47 (14–78)	NR^*^	Plate	Iliac crest/local autograft	91.7% (22/24)	35.7% (10/28)	42.9% (12/28)	MEPS: 80 (25–100)
Lee et al., 2020	USA	Case series	Diaphysis nonunion	19	51.7 (28–81)	No more than 2	Plate	Tricalcium phosphate	82% (14/17)	52.9% (9/17)	17.6% (3/17)	No functional score reported
Zha et al., 2021	China	Case report	Chronic intercondylar fracture	1	26	2	Plate	Iliac crest	100%	0	0	MEPS: 100
Ceynowa et al., 2018	Poland	Case report	Proximal nonunion	1	55	6	Plate	NR	100%	100%(1/1)	200% (2/1)	No functional score reported
Arikan et al., 2018	Turkey	Case series	Diaphysis nonunion	6	45 (24–68)	1.8 (0.5–3.5)	Plate	Iliac crest	100%	88.3% (5/6)	0	Constant-Murley score: 62.3 (35–75)
Xiao et al., 2016	China	Case series	Infected diaphysis nonunion	7	40.9 (26–61)	3 (2–4)	Plate as external fixator	None	100%	28.6% (2/7)	0	DASH: 3.2 (0–13.4)
Wang et al., 2013	China	Case series	Total brachial plexus avulsion injury	35	NR	4.2(3–4.5)	Plate	None	NR	NR	NR	No functional score reported
Erden et al., 2012	Turkey	Case series	Diaphysis nonunion	5	49.2 (37–60)	1.78 (1.2–2.4)	Plate	Fibular autograft	100%	0	0	Constant-Murley score: 88 (80–95)
Beltran et al., 2010	USA	Case report	Disarticulated elbow	1	23	2.5	Plate	None	100%	0	0	No functional score reported
Sugun et al., 2009	Turkey	Case series	Amputation	7	18.7	4.3	Plate/K-wire/external fixator	None	NR	UC^**†**^	UC	Chen’s classification II: 2, III: 3, IV: 2
Bassiony et al., 2009	Egypt	Case series	Infected nonunion	8	40.6 (23–60)	2 (1–3)	External fixator	None	100%	87.5% (7/8)	0	No functional score reported
Dhar et al., 2008	India	Case series	Infected nonunion	11	38 (17–59)	2.9 (1–4)	External fixator	None	100%	173% (19/11)	NR	No score name: Excellent: 7, Good: 4
Brennan et al., 2008	USA	Case series	Diaphyseal nonunion	11	45.2 (18–69)	1.3 (0.5–3)	Plate	None/autogenous bone graft/demineralized bone matrix	100%	0	0	No functional score reported
Rose et al., 2007	India	Case series	Infected nonunion	2	31.5 (18–45)	2.5 (2–3)	External fixator	Iliac crest	50% (1/2)	150% (3/2)	50% (1/2)	Stewart and Hundley criteria: Fair: 1, Poor: 1
Sabapathy et al., 2007	India	Case series	Amputation	3	17.3(12–25)	7(6–7.5)	Plate	None	100%	66.7% (2/3)	66.7% (2/3)	Chen’s classification II: 1, III: 2
Khan et al., 2005	Pakistan	Case series	Diaphyseal nonunion	15	(30–80)	NR	Plate	Cancellous graft	100%	20% (3/15)	0	No score name: Excellent: 6, Good: 7, Poor: 2
Yu et al., 2003	China	Case series	Total brachial plexus avulsion injury	3	25 (18–30)	17.7 (13–20)	NR	NR	NR	NR	NR	No functional score reported
Chuang et al., 2001	China	Case series	Amputation	10	NR	8 (7–12); Shortening of 5 patients not available	NR	NR	NR	UC	UC	No functional score reported
Luccia et al., 2000	Brazil	Case report	Disarticulated elbow	1	25	3	Periosteal Vycril sutures	None	100%	0	0	No functional score reported
Patel et al., 2000	UK	Case series	Nonunion	3	40 (21–73)	6.3 (4–8)	External fixator	None	100%	133% (4/3)	0	No score name: 38.3 (34–44)
Windhager et al., 1995	Austria	Case series	Malignant bone and soft-tissue tumor	12	35.2 (5–68.5)	NR	NR	None	NR	UC	UC	Enneking criteria: Excellent: 1, Good: 6, Fair: 3 (Dead: 2)

*NR Not report; *† UC* Unable to calculate

### Synthesis of results

#### Indications of acute shortening

**Fig. 2:**
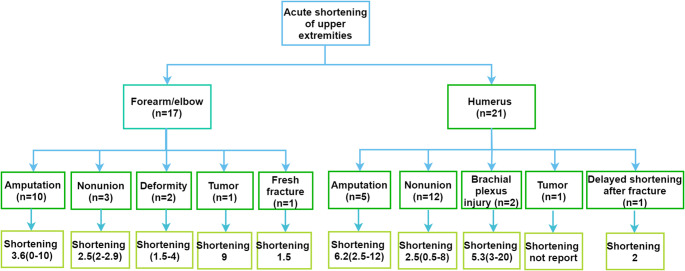
The included studies’ diagnosis and shortening length (cm) of the upper extremity

The diagnosis and shortening length for the forearm/elbow and humerus are shown in Fig. 2. For the forearm/elbow shortening studies, indications included amputation (*n* = 10) [6, 7, 9, 18, 34, 38, 42, 46, 49, 50], fracture nonunion(*n* = 3) [19, 20, 28], forearm deformity (*n* = 2) [25, 51], tumor (*n* = 1) [30] and acute fracture (*n* = 1) [21]. For the studies reporting on humeral shortening, the indications were fracture nonunion (*n* = 12) [12, 24, 26, 27, 29, 31, 33, 37, 39–41, 43], amputation (*n* = 5) [7, 36, 42, 46, 47], total brachial plexus invasion injury (*n* = 2) [32, 44], tumor (*n* = 1) [48] and delayed shortening after 2 months from sustaining the fracture (*n* = 1) [35].

#### Shortening length

Regarding the shortening length, the maximum shortening length for the forearm/elbow in amputation patients was 10 cm, as reported by Sabapathy et al. [42]

In a patient diagnosed with basal cell carcinoma of the elbow, the forearm and elbow were shortened by 9 cm after tumor resection, followed by vessel re-anastomosis [30]. The reported shortened length of the forearm/elbow was shorter in the other indications (nonunion, deformity and fracture), ranging from 1.5 to 4 cm.

Similarly, the maximum shortening length in the amputations of the humerus reached 12 cm in the study by Chuang et al. [46]. However, the largest shortening of the humerus (20 cm) was performed in 2 cases of associated brachial plexus injury to allow the direct anastomosis of the median and ulnar nerves to the contralateral normal C7 nerve route [44]. The shortening length was smaller for nonunion and fracture fixation of the humerus. Nevertheless, acute shortening of 7 cm and 8 cm were reported by Patel et al. [12]

#### Surgical information and clinical outcomes

After acute shortening, various implants were used for fixation, including pins, K-wires, cerclage wire, external fixators and plates [6, 7, 9, 12, 18–21, 24–44, 46–51]. Similarly, bone grafts, including autograft and bone substitutes, were used in nonunion patients [19, 21, 24, 27–31, 35, 40, 41, 43, 51]. The union rate was high, ranging between 82% and 100%, excluding the series of two cases reported by Rose et al. [41], of which one developed a refracture. The patient with bilateral forearm amputation reported by Chen et al. developed a nonunion of both sides, given a union rate of 0% [49]. In fracture nonunion treatment (3 cases for the forearm and 116 for the humerus), the overall union rate was 95% [12, 19, 20, 24, 26–29, 31, 33, 37, 39–41, 43]. 

The complication and reoperation rate ranged from 0 to 300% [12, 19–21, 24–31, 33–37, 39–43, 47–49, 51]. The maximum complication rate of 300% was identified in the case report of Chen at al of bilateral forearm shortening [49]. Complications encountered included infection, recurrent deformity, soft tissue necrosis, complex regional pain syndrome, rupture of the anastomosis vessel, nonunion/delayed union, contracture of the elbow joint, nerve injury, hematoma, dysesthesia/depression, pain, metal allergy, pin/implant loosening and refracture [12, 18, 24–26, 29, 31, 33, 37, 39, 41–43, 49]. However, in several studies [6, 7, 9, 18, 32, 38, 44, 46, 48], the complications and/or reoperations were described vaguely or not reported (Tables 1 and 2).

For patients undergoing replantation (forearm and humerus), Chen’s classification was predominantly used to assess functional outcomes [6, 7, 9, 18, 34, 38, 42, 50], with the exception of two studies that utilized descriptive metrics including range of motion (ROM), muscle strength, etc [46, 49]. In the studies of fracture nonunion and acute/delayed fractures, a variety of clinical scoring systems were utilized, such as the Mayo Elbow Performance Score (MEPS) [31, 35], the Disabilities of the Arm, Shoulder and Hand score (DASH) [19, 33], Constant-Murley score [24, 27] and Stewart and Hundley criteria [41], alongside several custom-developed scoring systems [12, 39, 43]. No clear evidence exists as to whether a specific length of shortening is associated with a poor outcome.

The postoperative range of motion following forearm shortening in acute fractures and nonunions is illustrated in Table 3 [19–21, 28]. A certain degree of limitations in supination and pronation were observed in adult patients [19–21] when the shortening was between 1.5 and 2.9 cm. Notably, despite the considerable shortening in several cases, no subsequent lengthening procedures were reported.

**Table 3:** Range of motion after forearm shortening in nonunion or fracture patients

Reference, first author, date	Diagnosis	No. of patients	Age (yr)	Length of shortening (cm)	Elbow ROM^*^ (°)		Forearm ROM(°)		Wrist ROM(°)	
					Injured side	Normal side	Injured side	Normal side	Injured side	Normal side
Srikanth et al., 2024	Nonunion	1	43	2	Extension:0 Flexion:140	Extension: NR^†^ Flexion: NR	Supination:70 Pronation: 50	Supination: NR Pronation: NR	Dorsi flexion: NR Volar flexion: NR	Dorsi flexion: NR Volar flexion: NR
Maheshwar et al., 2015	Nonunion of ulna	1	7	2.5	Extension: Normal Flexion: Normal	Extension: Normal Flexion: Normal	Supination: Normal Pronation: Normal	Supination: Normal Pronation: Normal	Dorsi flexion: Normal Volar flexion: Normal	Dorsi flexion: Normal Volar flexion: Normal
Sharma et al., 2004	Ulnar nonunion	1	19	2.9	Extension:0 Flexion:144	Extension: −2 Flexion:144	Supination:64 Pronation:52	Supination:70 Pronation:70	Dorsi flexion:72 Volar flexion: 82	Dorsi flexion:72 Volar flexion:84
Chauhan et al., 1995	Acute comminuted fracture	1	51	1.5	Extension: Normal Flexion: Normal	Extension: Normal Flexion: Normal	Supination:80 Pronation: 45	Supination:90 Pronation:90	Dorsi flexion: 60 Volar flexion:60	Dorsi flexion: 80 Volar flexion:80

*ROM Range of motion; *NR* Not report

## Discussion

Acute shortening of the upper extremity could be performed in a single stage for segmental bone loss or large soft tissue defects to avoid complex soft tissue reconstruction. There was no similar review systematically analyzing the indications and shortening distance of acute shortening surgeries of the upper limbs in the literature. Through the rigorous literature retrieval and data synthesis, this scoping review found that the main indications for acute shortening were amputation and fracture nonunion.

The forearm was most commonly shortened in amputation patients, whereas the humerus was shortened in nonunions. Other reported indications included tumor resection, deformity correction, comminuted fracture and brachial plexus injury. Acute shortening was considered essential in replantation surgery; the reported shortening length was usually larger than in other procedures. An average shortening of 2.5 cm was reported in both forearm and humerus nonunion patients.

Aggressive shortening was recommended in the setting of amputations for tension-free neurovascular anastomosis, considering the survivorship of the extremity as the priority. Malt et al. recommended 5–7 cm of bone shortening in such cases so that blood vessels and peripheral nerves would be amenable to end-to-end suturing [52]. Leclère et al. claimed that bone shortening of the forearm by 5–8 cm was well tolerated [18]. Sabapathy et al. stated that bone shortening of up to 10 cm of either the humerus or the forearm had not produced any functional or cosmetic problems [42]. According to Chuang et al., bone shortening in the forearm and humerus to address traction avulsion amputations should be at least 5 cm and 7 cm, respectively [46]. It is generally accepted that bone shortening was partially responsible for good results [6, 9, 49]. 

Acute shortening for nonunions was mainly performed at the humerus. One of the advantages was reducing the need and/or amount of autogenous bone graft and contouring of the nonunion ends to allow internal fixation with compression [29, 39, 53]. Another advantage was avoiding secondary surgery in infected nonunion patients with aggressive debridement of the infected bone, shortening of the bone segment and compression by an external fixator [33, 37, 41]. 

We identified certain controversies between the different series regarding the recommended shortening length in these clinical scenarios. The main concern was muscle weakness caused by decreased lever-arm of the extremity muscles [16]. Using a computational model, a significant reduction of the triceps muscle force after > 2 cm shortening of the distal humerus was identified [17]. However, the results might differ in vivo because of muscle adaptation and differences between patients’ demands. Unfortunately, muscle force was not reported in any of the nonunion humerus shortening studies [12, 24, 26, 27, 29, 31, 33, 37, 39–41, 43]. 

Shortening within 2 cm was generally accepted to be well tolerated functionally [29, 31, 54]. Bone loss less than 3–4 cm was reported to lead to good radiological and clinical results [5, 24, 33, 37]. Other authors advocated that shortening up to 5 cm would not interfere with the cosmetic appearance or daily function [11, 39]. Wang et al. recommended that the humerus should be shortened by no more than 5 cm in adults and no more than 3 cm in children, or else shortening more than 12–14% of its original length is expected to cause malfunction of the triceps and biceps muscles and cosmetic problems [10, 32]. 

Shortening the proximal humerus requires additional attention, especially when the deltoid tuberosity is involved, which might lead to the dislocation of the shoulder joint [26]. Shortening at the supracondylar region was also challenging as it requires the reconstruction of the humeral fossae for the coronoid, radial head and olecranon [31]. 

Shortening the forearm may affect muscle strength and cause restriction of the forearm rotation. In the cadaveric study conducted by Barinaga et al., the author observed that increased shortening of the radius and ulna led to progressively more substantial reductions in both pronation and supination range of motion [14]. Supination decreased more between 2 and 4 cm of shortening than the pronation. Another biomechanical study concluded that shortening of the middle and distal third of the forearm might lead to more loss of rotation than shortening of the proximal third [15]. 

Shortening the forearm due to a nonunion or an acute fracture has been recorded seldomly [19–21, 28]. In the four cases identified in this review, one child had no deficit of pronation-supination of the forearm and grip strength. In contrast, three adults developed limited pronation/supination to varying degrees (Table 3).

After shortening, a variety of implants and bone-grafts were used in the enrolled patients. Plates were chosen most frequently for fixation, followed by external fixator. The necessity of bone grafting and the choice of grafting materials remained controversial, particularly in the treatment of fracture nonunion. However, it should be noted that shortening procedures theoretically reduce the requirement for bone grafting. Even when grafting was performed, the volume of graft material needed was significantly decreased [2, 4, 5]. 

Clinical functional outcomes are influenced by multifactorial determinants, primarily attributed to the initial injury characteristics and underlying pathology‌. Thus, it’s difficult to identify the relationship between shortening length and patients’ outcomes. Theoretically, minimization of shortening length should be pursued based on clinical needs to eliminate potential confounding effects. [14, 15

The contributions of this review are clarifying the main indications of acute shortening of upper limb and identifying the tolerable shortening distances for different diagnosis. Yet, our scoping review has some limitations. Only studies published in English were included, leading to the possibility of missing acute shortening articles published in other languages. Only peer-reviewed published studies were included, which might result in contemporary research missing, such as conference abstracts. The quality of evidence of the retrieved studies was low as only small retrospective non-controlled case series and case reports were identified. Thus, no definite conclusions could be drawn. Instead, a descriptive summary of available evidence is provided.

## Conclusions

This scoping review summarizes currently available evidence on the acute shortening procedures of the upper extremities. Current literature suggests that the main indications for acute upper limb shortening were amputations during the reimplantation and fracture nonunion. It is generally accepted that bone shortening was critical to success in the setting of amputations. Shortening up to 10 cm of the forearm and up to 12 cm of the humerus were reported in the reimplantation procedures with good results. In acute fracture and fracture nonunion, the maximum shortening length, without significant functional deficit or cosmetic concerns, was 2.9 cm at the forearm and 8 cm at the humerus.

## Electronic supplementary material

Below is the link to the electronic supplementary material.

Supplementary Material 1

## Data Availability

No datasets were generated or analysed during the current study.
