# Supplementary material for: Acute shortening of upper extremity in orthopaedic patients: a scoping review
Source: Eur J Trauma Emerg Surg. 2025 Jun 11;51(1):225. doi: 10.1007/s00068-025-02904-9 (PMC12159111; doi:10.1007/s00068-025-02904-9)
Supplement: Supplementary file 1 — Supplementary Material 1 [file 68_2025_2904_MOESM1_ESM.docx]

**Search strategy of databases**

Pubmed:

(((((((deformity osteotomy) OR (tumor resection)) OR (fracture nonunion)) OR (infection debridement)) OR (amputations)) OR (Mangled extremity injuries)) OR (Acute bone loss)) AND (((acute shortening) OR (shortening)) AND ((((upper limb) OR (upper extremity)) OR (forearm)) OR (humerus))) only in English

Web of science:

1. upper limb (Topic) and Upper Extremity (OR – Search within topic) and Humerus (OR – Search within topic) and Forearm (OR – Search within topic) and Shortening (Search within topic) and Acute Bone Loss (Search within topic) and Preprint Citation Index (Exclude – Database)
2. upper limb (Topic) and Upper Extremity (OR – Search within topic) and Humerus (OR – Search within topic) and Forearm (OR – Search within topic) and Shortening (Search within topic) and Mangled Extremity Injuries (Search within topic) and Preprint Citation Index (Exclude – Database)
3. upper limb (Topic) and Upper Extremity (OR – Search within topic) and Humerus (OR – Search within topic) and Forearm (OR – Search within topic) and Shortening (Search within topic) and Amputation (Search within topic) and Preprint Citation Index (Exclude – Database) and English (Languages)
4. upper limb (Topic) and Upper Extremity (OR – Search within topic) and Humerus (OR – Search within topic) and Forearm (OR – Search within topic) and Shortening (Search within topic) and Infection Debridement (Search within topic) and Preprint Citation Index (Exclude – Database) and English (Languages)
5. upper limb (Topic) and Upper Extremity (OR – Search within topic) and Humerus (OR – Search within topic) and Forearm (OR – Search within topic) and Shortening (Search within topic) and Fracture Nonunion (Search within topic) and Preprint Citation Index (Exclude – Database) and English (Languages)
6. upper limb (Topic) and Upper Extremity (OR – Search within topic) and Humerus (OR – Search within topic) and Forearm (OR – Search within topic) and Shortening (Search within topic) and Deformity Osteotomy (Search within topic) and Preprint Citation Index (Exclude – Database) and English (Languages)
7. upper limb (Topic) and Upper Extremity (OR – Search within topic) and Humerus (OR – Search within topic) and Forearm (OR – Search within topic) and Shortening (Search within topic) and Tumor Resection (Search within topic) and Preprint Citation Index (Exclude – Database) and English (Languages)

Embase:

1. ('upper limb'/exp OR 'upper limb' OR 'upper extremity'/exp OR 'upper extremity' OR 'humerus'/exp OR 'humerus' OR 'forearm'/exp OR 'forearm') AND 'shortening' AND ('amputation'/exp OR 'amputation') AND [english]/lim
2. ('upper limb'/exp OR 'upper limb' OR 'upper extremity'/exp OR 'upper extremity' OR 'humerus'/exp OR 'humerus' OR 'forearm'/exp OR 'forearm') AND 'shortening' AND 'fracture nonunion' AND [english]/lim
3. ('upper limb'/exp OR 'upper limb' OR 'upper extremity'/exp OR 'upper extremity' OR 'humerus'/exp OR 'humerus' OR 'forearm'/exp OR 'forearm') AND 'shortening' AND 'tumor resection' AND [english]/lim

Cochrane library

#1 (upper limb):ti,ab,kw OR (upper extremity):ti,ab,kw OR (humerus):ti,ab,kw OR (forearm):ti,ab,kw (Word variations have been searched)

#2 (shortening):ti,ab,kw OR (acute shortening):ti,ab,kw (Word variations have been searched)

#3 #1 and #2

#4 (Acute bone loss):ti,ab,kw OR (Mangled extremity injuries):ti,ab,kw OR (amputation):ti,ab,kw AND (infection debridement):ti,ab,kw OR (fracture nonunion):ti,ab,kw (Word variations have been searched)

#5 (deformity osteotomy):ti,ab,kw OR (Tumor resection):ti,ab,kw (Word variations have been searched)

#6 #4 or # 5

#7 #3 and #6 in English
